# Supplementary material for: Plastic Food Packaging from Five Countries Contains Endocrine- and Metabolism-Disrupting Chemicals
Source: Environ Sci Technol. 2024 Mar 5;58(11):4859–71. doi: 10.1021/acs.est.3c08250 (PMC10956434; doi:10.1021/acs.est.3c08250)
Supplement: Supplementary file 1 — es3c08250_si_001.pdf [file es3c08250_si_001.pdf]

Supporting Information for

Plastic food packaging from five countries contains endocrine and metabolism disrupting chemicals

Sarah Stevens,[1] *Molly McPartland,*[1] *Zdenka Bartosova,* [1] *Hanna Sofie Skåland,*[1] *Johannes Völker,* [1,2] *Martin Wagner*[1]

[1] Norwegian University of Science and Technology (NTNU), Department of Biology, 7491 Trondheim, Norway

[2] present address: Innovative Environmental Services (IES) Ltd, Benkenstrasse 260, 4108 Witterswill, Switzerland.

[*] corresponding authors: sarah.stevens@ntnu.no and martin.wagner@ntnu.no

This file includes:

S1 Supporting methods and materials

S2 Supporting results and discussion

Figures S1 to S10

Tables S1 to S13

Supplementary references

Other supplementary materials for this manuscript include the following:

Table S14-S15

Mass spectral data of all samples can be assessed under https://doi.org/10.18710/LZNLFX

S1 Supporting methods and materials

S1.1 Determination of polymer type

Where not stated on the packaging, the polymer types of the FCA were determined with Fourier-transformed infrared spectroscopy (Cary 610-FTIR and Resolutions Pro software, Agilent) in attenuated total reflectance and transmission mode for the expanded polymers (LDPE 8, PS 5a and b). The polymer type was determined for the inside and outside of the samples. Spectra were acquired with a resolution of 4 cm^{-1} and 32 scans per spectrum and matched to the library of Primpke et al.[1] Differentiation between HDPE and LDPE products was done by determining the melting point with differential scanning calorimetry (TGA/DSC 1 thermogravimeter, Mettler Toledo) in the temperature range of 25–180 °C. The measurements were carried out in an N_2 atmosphere with a 50 mL min^{-1} flow rate and a 10 K min^{-1} heating rate.

S1.2 Reporter gene assays

The CALUX cell lines were cultured at 37 °C and 5% CO_2 in growth medium consisting of DMEM/F-12 (GlutaMAX, Gibco, 31331093) supplemented with 5% fetal bovine serum (FBS, Sigma Aldrich, F9665), 5.4 mL non-essential amino acids (Sigma Aldrich, M7145), 1% penicillin/streptomycin (Biowest, L0022) and 0.4% geneticin (Sigma Aldrich, A1720). All cell lines were used between passages 3 and 15 and at 70–90% confluency. For the ERα assay, the growth medium was changed to assay medium (DMEM/F-12 with 5% charcoal-stripped FBS (GlutaMAX, Gibco, 11580546), 5.1 mL non-essential amino acids, and 1% penicillin/streptomycin) 2 d prior to the experiments to avoid that the steroids present in normal FBS interfere with the receptor. All assays were conducted with assay medium in 384-well plates (CellStar 781098, Greiner Bio-One). The outer line of the plates (two lines for the Anti-AR assay) contained medium without cells to prevent edge effects on cell growth. 5000 cells $well^{-1}$ were seeded 24–28 h prior to the 23-h exposure.

Table S1. Reference compounds used in the bioassays.

Receptor	Reference compound	CAS	Supplier	Concentration range [mol L-1]
PXR	nicardipine hydrochloride	54527-84-3	Sigma-Aldrich	9.4×10^{-9} - 4.8×10^{-6}
PPARγ	rosiglitazone	122320-73-4	Sigma-Aldrich	3.7×10^{-10} - 3×10^{-6}
ERα	17-beta-estradiol	50-28-2	Sigma-Aldrich	1.9×10^{-13} - 1.0×10^{-10}
AR	flutamide	13311-84-7	Sigma-Aldrich	1×10^{-8} - 3×10^{-5}

S1.3 Chemical analysis

For chromatographic separation a 38-min linear gradient program was used (Table S1) and compounds eluting between 1.5 and 36 min were included in the analysis. The flow rate was set to 0.2 mL min^{-1}, the column temperature was maintained at 55°C and the injection volume was 2 µL. After each sample a 7 min wash step with 100% methanol at 0.35 mL min^{-1} was included, followed by a 3 min equilibration to the initial column conditions. The collision energy ramped from 15 to 45 V, and the scan time was 0.3 s (details in Table S3). The analysis included all samples, PBs 1–4, the solvents used during extraction (methanol evaporated to DMSO), and **7** quality controls (pool of all PUR and PVC or PP, PE, PET, and PS samples).

Data treatment and compound identification was performed with the software Progenesis QI (Nonlinear Dynamics, version 3.0). The retention times were automatically aligned to one of the quality controls (pool of all samples) selected by the software. Peak picking was performed with automatic sensitivity to detect "fewer" peaks. The minimum chromatographic peak width was set to 0.05 min and the fragment sensitivity was set to 0.2% of the base peak. The program searched for common adducts (M+H, 2M+H, M+2H, M+H-H_2O, M+Na, and 2M+Na), and deconvoluted the mass spectra. The resulting list of chemical features was exported to Microsoft Excel for Windows (Version 2021-2306).

The R package "pheatmap" [2] was used for the hierarchical cluster analysis (Euclidian distances) of the chemical features (Figure 2C and 4I). Euler diagrams were produced with the R package "eulerr" (Figure 3 and S4).[3]

Table S2. Chromatographic parameters for the nontarget chemicals analysis.

Retention time (min)	Mobile phase A (%, water with 0.1% formic acid)	Mobile phase B (%, methanol with 0.1% formic acid)
0	80	20
0.5	80	20
29	5	95
36	5	95
36.1	0	100
38	0	100

Table S3. Mass spectrometer parameters for the nontarget chemicals analysis.

Parameter	Value
Capillary (kV)	2.8
Source temperature (°C)	120
Sampling cone voltage (V)	30
Source offset voltage (V)	50
Desolvation temperature (°C)	500
Cone gas flow (L h^{-1})	500
Desolvation gas flow (L h^{-1})	900
Nebulizer gas flow (bar)	6

S1.4 Compound identification, ToxCast and PubChem search

Identification was done with the Metascope algorithm in Progenesis QI. For the *in silico* fragmentation, four databases were used as previously described.[4] In addition, we constructed a fifth database containing the plastic chemicals described by Wiesinger et al.[5] For their PlasticMap database, we converted the available 10 547 CASRNs to SMILES-codes (n = 6631) using the EPA CompTox batch search.[6] The SMILES codes were then converted to CIDs (n = 6423) using the PubChem Identifier Exchange service [7]. CIDs were uploaded to PubChem to retrieve the chemical structures (n = 6414) as a sdf file which was subsequently used for the *in silico* fragmentation search.

Among the tentatively identified compounds 304 were present in the ToxCast data based on matching InChlKeys, IUPAC names or common names. We extracted their activity concentrations 50 (AC_{50}) in 45 ToxCast assays that matched the receptors analyzed here (Table S4). ToxCast assays were selected based on the receptor gene names. The AC_{50} values were compared to the compound's highest peak area and ranked based on the AC_{50}/area ratio, for all compounds with a peak area >100 (Table S9). A Spearman rank correlation between number of compounds activating a certain receptor and the samples effect concentrations was calculated with GraphPad Prism (9-10).

For the tentatively identified compounds among the ten features with the highest abundance per sample, an additional search for toxicity and usage information was done on PubChem,[8] and in the supplementary information from Wiesinger et. al. and Groh et al.[5,9]

Table S4. Selected ToxCast assays used to retrieve information about PXR, PPARγ, estrogenic and anti-androgenic activity.

Receptor	Assay name in ToxCast	Mode
**PXR**	ATG_PXRE_CIS_up	agonist
	ATG_PXR_TRANS_up	agonist
	NVS_NR_hPXR	agonist
	TOX21_PXR_Agonist	agonist
	ATG_PXRE_CIS_dn	antagonist
	ATG_PXR_TRANS_dn	antagonist
**PPARγ**	ATG_PPRE_CIS_up	agonist
	ATG_PPARg_TRANS_up	agonist
	NVS_NR_hPPARg	agonist
	OT_PPARg_PPARgSRC1_0480	agonist
	OT_PPARg_PPARgSRC1_1440	agonist
	TOX21_PPARg_BLA_Agonist_ratio	agonist
	TOX21_PPARg_BLA_antagonist_ratio	antagonist
	ATG_PPARg_TRANS_dn	antagonist
	ATG_PPRE_CIS_dn	antagonist
**ERα**	ACEA_ER_80hr	agonist
	ATG_ERE_CIS_up	agonist
	ATG_ERa_TRANS_up	agonist
	NVS_NR_hER	agonist

Receptor	Assay name in ToxCast	Mode
**ERα**	OT_ER_ERaERa_0480	agonist
	OT_ER_ERaERa_1440	agonist
	OT_ERa_EREGFP_0120	agonist
	OT_ERa_EREGFP_0480	agonist
	TOX21_ERa_BLA_Agonist_ratio	agonist
	TOX21_ERa_LUC_VM7_Agonist	agonist
	TOX21_ERa_LUC_VM7_Agonist_10nM_ICI182780	agonist
	ATG_ERa_TRANS_dn	antagonist
	ATG_ERE_CIS_dn	antagonist
	TOX21_ERa_BLA_Antagonist_ratio	antagonist
	TOX21_ERa_LUC_VM7_Antagonist_0.5nM_E2	antagonist
	TOX21_ERa_LUC_VM7_Antagonist_0.1nM_E2	antagonist
**AR**	ATG_AR_TRANS_dn	antagonist
	NVS_NR_hAR	antagonist
	OT_AR_ARELUC_AG_1440	antagonist
	OT_AR_ARSRC1_0480	antagonist
	OT_AR_ARSRC1_0960	antagonist
	TOX21_AR_BLA_Antagonist_ratio	antagonist
	TOX21_AR_LUC_MDAKB2_Antagonist_10nM_R1881	antagonist
	TOX21_AR_LUC_MDAKB2_Antagonist_0.5nM_R1881	antagonist
	ACEA_AR_antagonist_80hr	antagonist
	ATG_AR_TRANS_up	agonist
	TOX21_AR_BLA_Agonist_ratio	agonist
	TOX21_AR_LUC_MDAKB2_Agonist	agonist
	TOX21_AR_LUC_MDAKB2_Agonist_3uM_Nilutamide	agonist
	ACEA_AR_agonist_80hr	agonist

S2 Supporting results and discussion

S2.1 Receptor activity and cytotoxicity

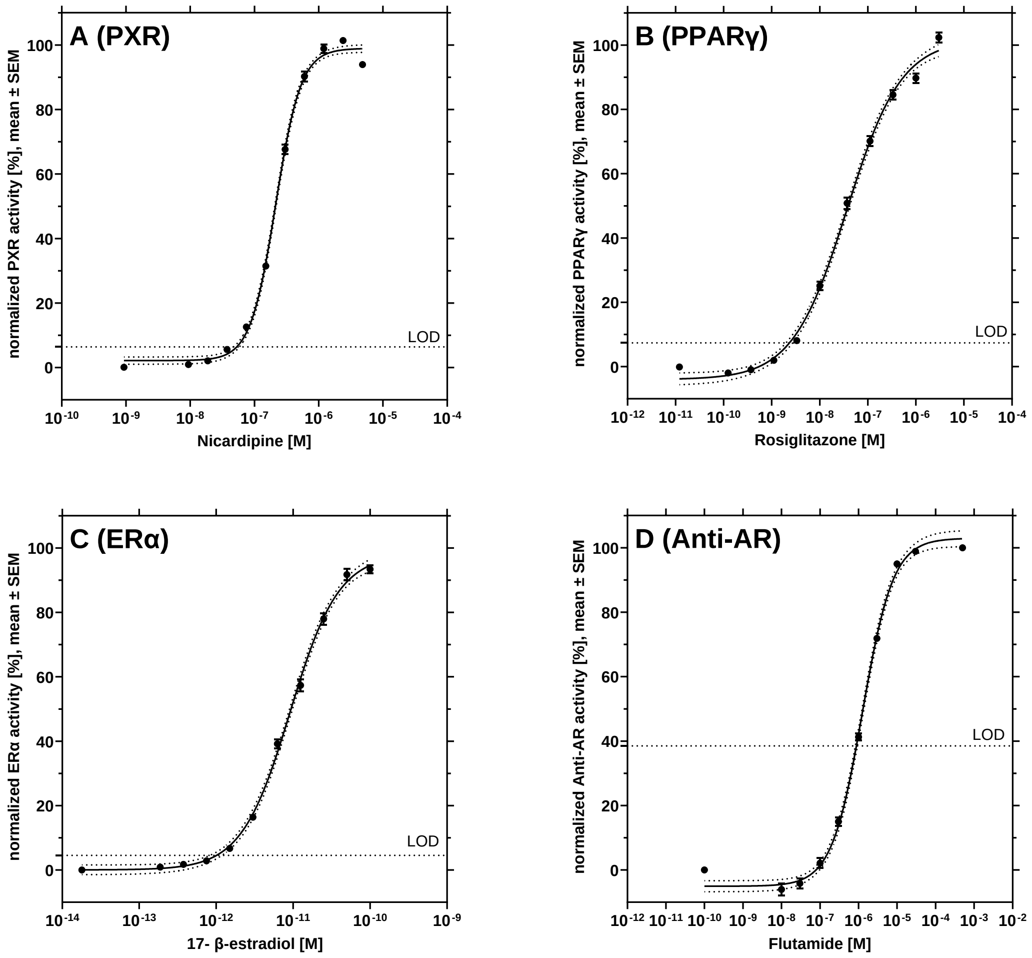

Figure S1. Dose-response relationships of the reference compounds used in the reporter gene assays. Nicardipine was used for the pregnane X receptor (PXR, A), rosiglitazone for peroxisome proliferator receptor gamma (PPARγ, B), 17-β estradiol for estrogen receptor alpha (ERα, C), and flutamide for the anti-androgenic activity (Anti-AR, D). n ≥ 60. Note: LOD = limit of detection

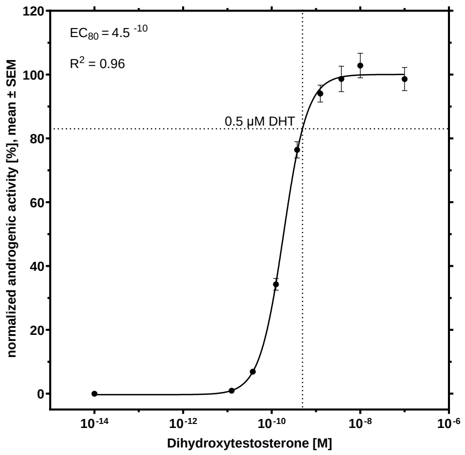

Figure S2. Dose-response relationship of the reference compound dihydrotestosterone (DHT) in the androgen assay. n = 12. Note: 0,5 µM DHT was used as background agonist in the antagonistic assays.

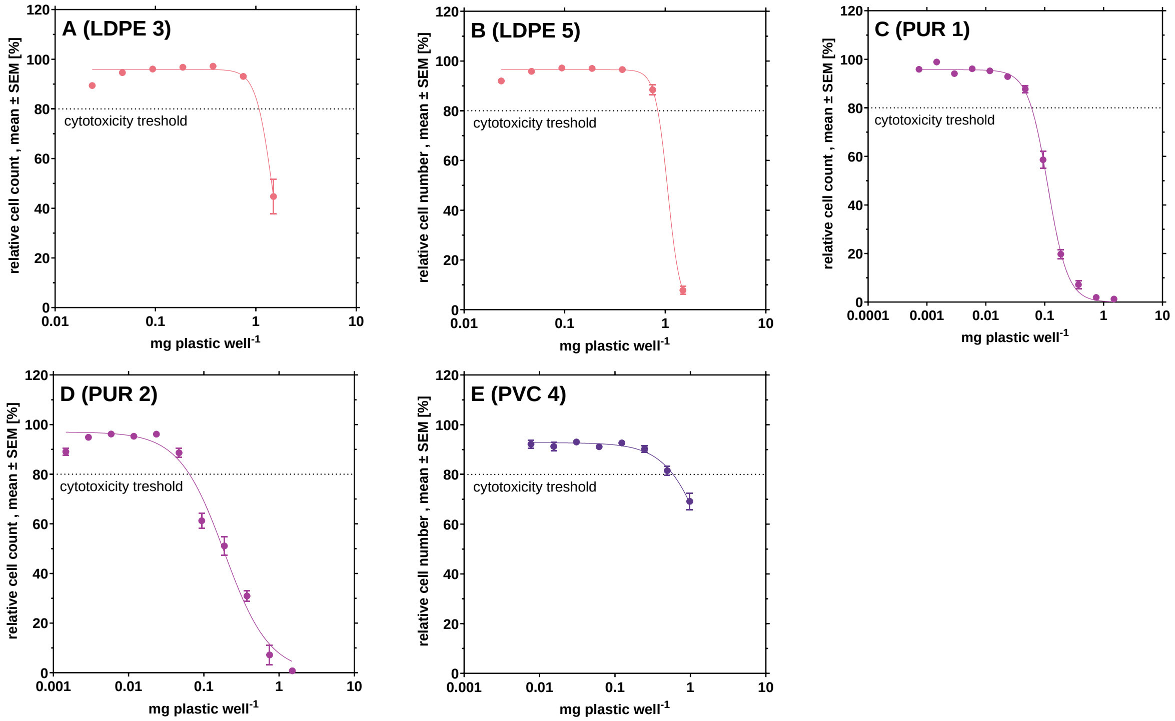

Figure S3**.** Cytotoxicity of samples LDPE 3 (A), LDPE 5 (B), PUR 1 (C), PUR 2 (D), PVC (4). Cytotoxicity is defined as a cell count <80% of the pooled negative and solvent controls. Cell count across receptors combined, for cytotoxic concentrations n ≥ 16, for non-cytotoxic concentrations n ≥ 48. Highest tested concentration of 1.5 mg plastic/well.

Cytotoxic chemicals were least prevalent in our samples (Figure S3): Five of the FCA extracts induced significant cytotoxicity, including two freezer bags (LDPE 3 and 5), two hydration bladders (PUR 1 and 2), and a cling film (PVC 4). The two PUR hydration bladders induced the

strongest cytotoxicity (EC_{20} 0.05 and 0.06 mg/well). Cytotoxicity correlates significantly with the number of chemical features (Figure S4) but this correlation is mostly driven by the two PUR samples. Nonetheless, cytotoxicity is mostly thought of as a result of non-specific effects that will accumulate when more chemicals are present in a sample.[10]

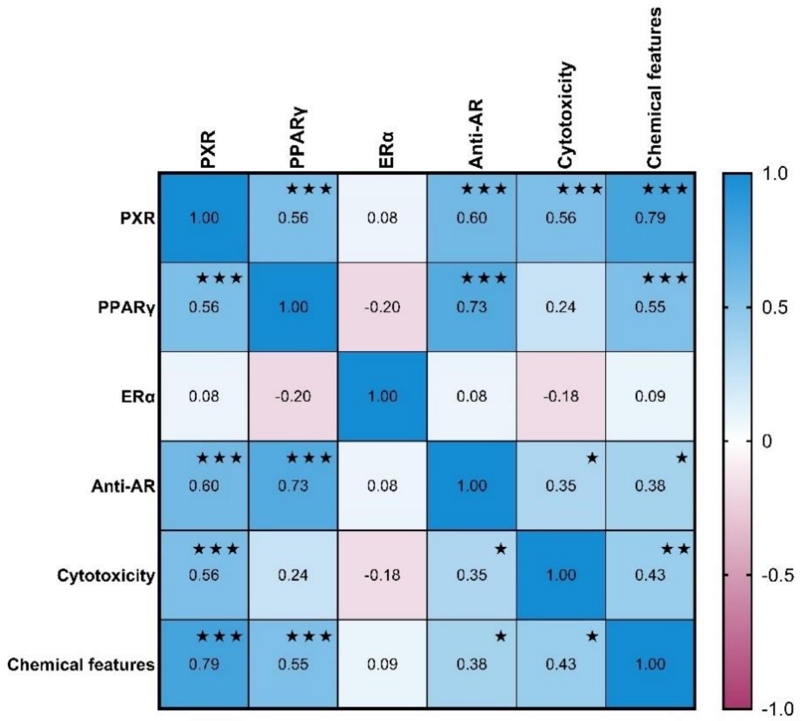

Figure S4. Correlation between biological effects ($EC_{20/50}$) and the number of chemical features detected in the plastic food contact articles. The values in the boxes represent Spearman's rank correlation coefficient, the asterisks indicate that the correlation was statistically significant (* $p < 0.05$, ** $p < 0.01$. *** $p < 0.001$).

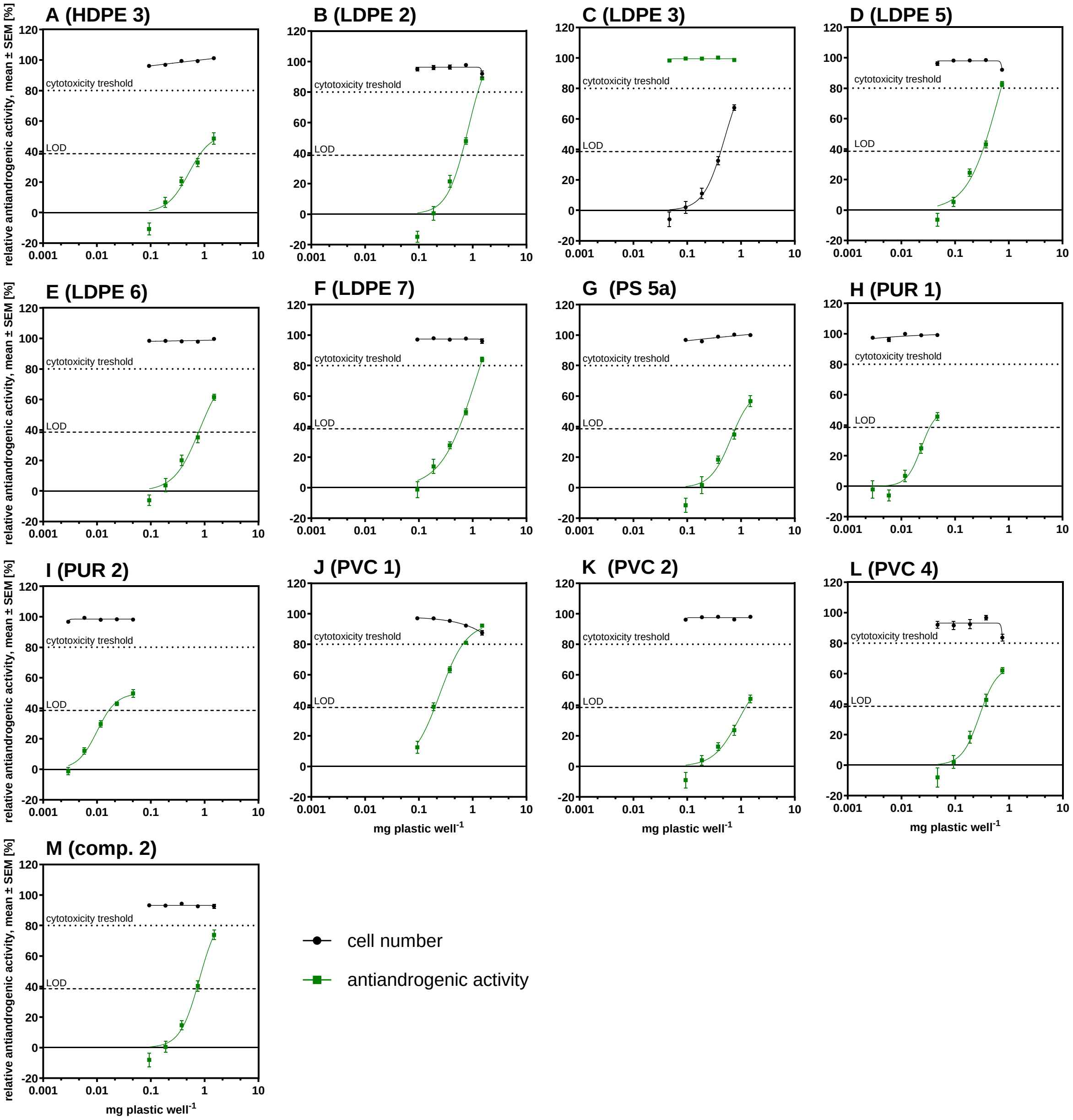

Figure S5. Dose-response relationships of the anti-androgenic samples with the corresponding cell count (normalized to controls). Data are derived from at least three independent experiments, with four technical replicates per concentration (n ≥ 12).

S2.2 Chemical analysis

Table S5. Distribution of chemical features across samples.

PE, PET, PP and PS samples			PUR and PVC samples		
Number of samples	Number of features shared between samples	Share of all 8819 features [%]	Sample number	Number of features shared between samples	Share of all 16 847 features [%]
1	3592	41.5	1	3659	21.7
2	1864	21.5	2	4827	28.7
3	985	11.4	3	3269	19.4
4	773	8.9	4	2583	15.3
5	416	4.8	5	1901	11.3
6	338	3.9	6	532	3.2
7	202	2.3	7 (all)	76	0.5
8	147	1.7			
9	88	1.0			
10	61	0.7			
11	28	0.3			
12	33	0.4			
13	29	0.3			
14	20	0.2			
15	11	0.1			
16	10	0.1			
17	12	0.1			
18	16	0.2			
19	5	0.1			
> 20	35	0.4			
29 (all)	0	0.0			

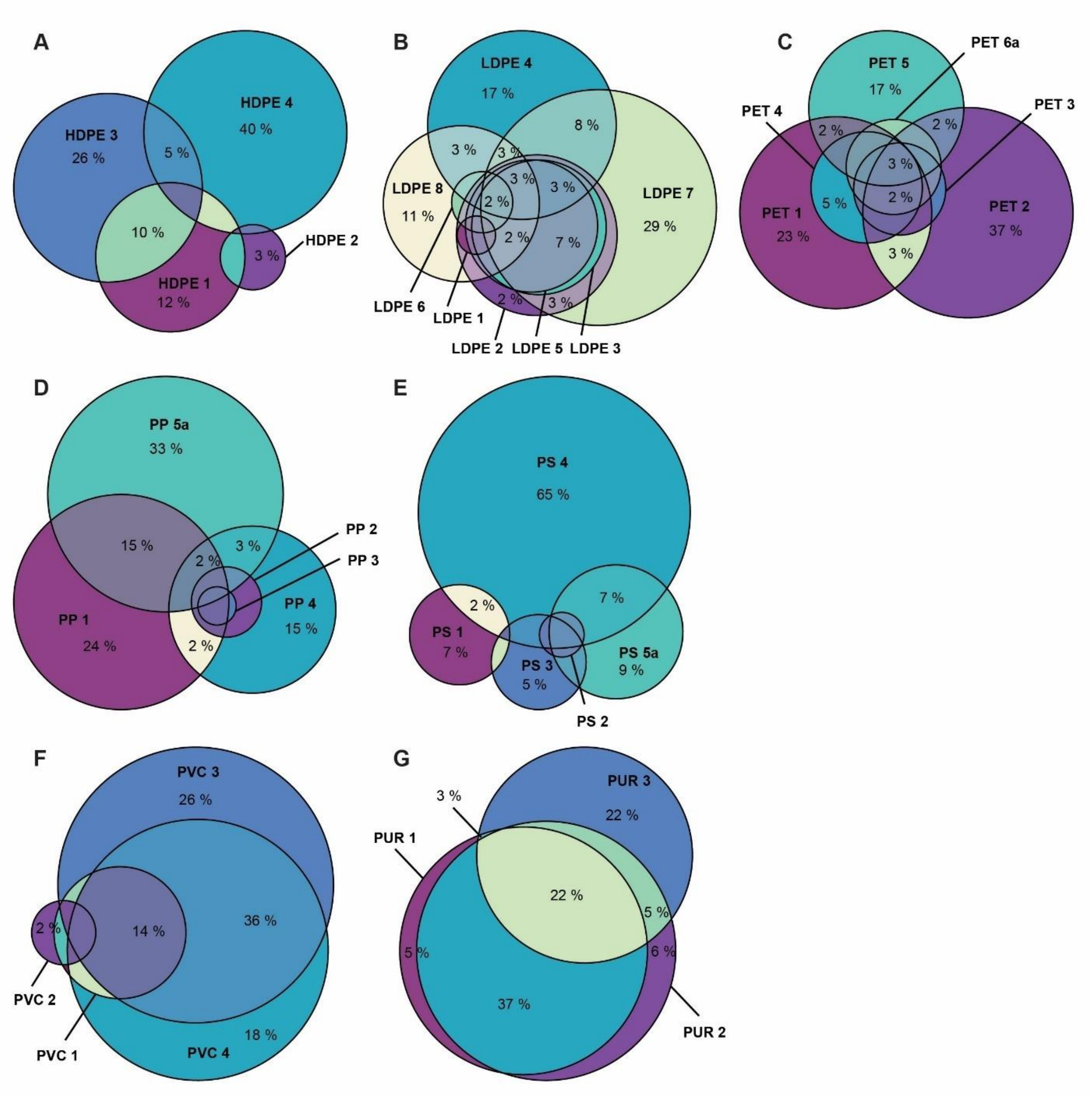

Figure S6. Overlap of chemical features in the samples made of the same polymer (A) HDPE, (B) LDPE, (C) PET, (D) PP, (E) PS, (F) PVC, (G) PUR and PUR-PE composite. An overlap of <1% is not shown.

Table S6. Chemical features in the FCAs made of the same polymer, total number, common features and features unique to a single product.

Polymer	N samples	All chemical features	Common chemical features	Unique chemical features
**HDPE**	4	616	3 (0.49%)	493 (80.0%)
**LDPE**	8	5494	2 (0.04%)	2817 (51.3%)
**PET**	6	1320	37 (2.8%)	965 (73.1%)
**PP**	5	2711	2 (0.07%)	1929 (71.1%)
**PS**	5	2284	41 (1.8%)	1864 (81.6%)
**PUR** [a]	3	13 004	2915 (22%)	4180 (32.1%)
**PVC**	4	12 683	179 (1.4%)	5738 (44.1%)

Note: [a] including the composite made of PUR and PE.

S2.3 Tentatively identified chemicals

Most of the chemicals were identified with the NORMAN Suspect List Exchange database (77% in the PE, PET, PP and PS samples and 91% in the PUR and PVC samples),[11] which is the most comprehensive database used in this study and includes about 70,000 compounds but is not specific to plastics.

Of the ten most abundant features per sample, we tentatively identified 69 compounds and retrieved use and toxicity information from Pubchem. Our analysis showed that 31 of these compounds were associated with plastics, including colorants, plasticizers, flame retardants, antioxidants, and other processing aids and further 12 are likely used in plastics (Table S8). Among the remaining features, 12 have no relevant use or toxicity data while 14 are not related to plastic and consist of cosmetics, pharmaceuticals, and fungicides among others (Table S8). Examples of plastic additives detected are erucamide (CAS 112-84-5) and octadecanamide (CAS124-26-5), which are used as colorants, fillers, and lubricants.[5] These substances were found with high abundance in the three LDPE freezer bags (LDPE 2, 3 and 5). Further, we detected the NIAS ethylene terephthalate cyclic trimer (CAS 7441-32-9) with high abundance in five of the six PET samples. This compound is known to occur in PET.[9]

Among the plastic additives, we detected several known toxic and/or persistent and bioaccumulative compounds with high abundances, such as triphenyl phosphate (TPP, CAS 115-

86-6), octrizole (CAS 3147-75-9), bis(1,2,2,6,6-pentamethylpiperidin-4-yl)decanedioate (CAS 41556-26-7), methyl 1,2,2,6,6-penta methyl- 4-piperidyl sebacate (CAS 82919-37-7) and ethyl 4-[(N-methylanilino)methylideneamino]benzoate (CAS 57834-33-0). The plasticizer and flame retardant TPP has bioaccumulative, persistent and endocrine disrupting properties[8] and interferes with all the receptors analyzed in this study.[12] TPP was detected in both PUR hydration bladders (PUR 1 and 2), while the other four compounds were detected in only one hydration bladder (PUR 2). Tributyl 2-acetyloxypropane-1,2,3-tricarboxylate (CAS 77-90-7) is another toxic plasticizer that was detected in an LDPE and PS sample (LDPE 2 and PS 5b) and is persistent, bioaccumulative, carcinogenic, and mutagenic. [8]

Table S7. Number of features and identified compounds detected in the samples.

Polymer set	Total number of chemical features	Tentatively identified chemical features	Tentatively identified compounds
PE, PET, PP, PS	8665	1760	1182
PUR, PVC	16 847	2377	1371
Total			2146

Table S8. Tentatively identified chemicals among the top ten features per sample with use and toxicity data retrieved from Groh et al. (2019) comprising plastic chemicals specific to food contact articles (A), Wiesinger et al. (2021) comprising intentionally added plastic chemicals (B), PubChem (C) and ToxCast (D).

CAS	Name	High abundance in sample	Plastic related	Use	Toxicity
23470-00-0	2,3-dihydroxypropyl hexadecanoate	LDPE_8	yes (B)	Cosmetics (C)	Toxic to aquatic life (C)
85391-47-5	2-O-(2-ethylhexyl) 1-O-undecyl benzene-1,2-dicarboxylate	HDPE 1, LDPE 4, PET 6b	tentative	Adhesive (C)	-
63753-10-6	2-hydroxy-5-nonylbenzaldehyde	HDPE 3	-	Used in metal extraction process (C)	corrosive, irritant, health hazard (C)
77-90-7	tributyl 2-acetyloxypropane-1,2,3-tricarboxylate	LDPE 2, PS 5b	yes (B)	Plasticizer, other processing aids, FCA (A, B)	Persistent, bioaccumulate, CMR, carcinogenic, mutagenic (B) PPARγ and PXR agonist (D)
112-84-5	erucamide	LDPE 2, LDPE 3, LDPE 5	yes (B)	Colorant, filler, lubricant, FCM (B)	Irritant (C). Not classified as of potential concern (B)
10436-08-5	icos-11-enamide	LDPE 2	yes (B)	FCM (B)	Not classified as of potential concern (B)
124-26-5	octadecanamide	LDPE 2, LDPE 3, LDPE 5, PS 1	yes (B)	Antistatic Agent, Colorant, Filler, Intermediates, Lubricant, FCM (B)	Not classified as of potential concern (B)
59227-89-3	laurocapram	LDPE 3	-	Skin conditioning (C)	Irritant (C). AR antagonist, PPARγ, ERα and PXR agonist (D)
94713-19-6	4-(4,7-dimethyl-2,3,3a,4,5,6,7,7a-octahydro-1H-inden-5-yl)-2-methylcyclohexan-1-ol	LDPE 3	-	-	-
5834-63-9	1,8,15,22-tetrazacyclooctacosane-2,9,16,23-tetrone	LDPE 4	yes (A, B)	-	-
621-70-5	2,3-di(hexanoyloxy)propyl hexanoate	LDPE 4, comp. 1	tentative	Lubricants and lubricant additives, textile manufacturing (C)	-

CAS	Name	High abundance in sample	Plastic related	Use	Toxicity
30899-62-8	2,3-diacetyloxypropyl dodecanoate	LDPE 4, comp. 1	yes (B)	plasticizer, biocide, colorant, filler, lubricant, other processing aids, cling films (B, C)	Not classified as of potential concern (B)
33599-07-4	2,3-diacetyloxypropyl octadecanoate	LDPE 4, comp. 1	yes (B)	Plasticizer in PVC (B)	Not classified as of potential concern (B)
56149-06-5	2,3-diacetyloxypropyl docosanoate	LDPE 4	-	-	-
537-40-6	2,3-bis[[(9Z,12Z)-octadeca-9,12-dienoyl]oxy]propyl (9Z,12Z)-octadeca-9,12-dienoate	LDPE 5	-	solvent, cosmetics (C)	-
25401-86-9	2-hexadecylphenol	LDPE 5	yes (C)	polycarbonate resins (C)	-
94291-86-8	8-(3,7-dimethyloct-6-enoxy)-8-methoxy-2,6-dimethyloctan-2-ol	LDPE 6	-	-	-
112-99-2	N-octadecyloctadecan-1-amine	LDPE 7, comp. 1, PP 1	tentative	Lubricant, hydrocarbon resin emulsion, sunscreen (C)	Irritant, toxic to aquatic life (C)
14130-06-4	docosan-1-amine	HDPE 4, PS 5a	-	Hair cosmetics, heat transfer fluids (C)	-
554-91-6	gentiobiose	HDPE 4	-	Biological molecule (C)	-
20198-87-2	N-benzyloctadecan-1-amine	HDPE 4	yes (C)	Thermoplastic elastomer (C)	-
510-75-8	12-hydroxy-11-methyl-6-methylidene-16-oxo-15-oxapentacyclo[9.3.2.15,8.01,10.02,8]heptadec-13-ene-9-carboxylic acid	HDPE 4	-	Fungicide (C)	-
119344-86-4	2-(dimethylamino)-2-[(4-methylphenyl)methyl]-1-(4-morpholin-4-ylphenyl)butan-1-one	PP 1	yes (B)	UV light absorber, paints and coatings (C)	Reproductive toxicity, harmful to aquatic life (C)

CAS	Name	High abundance in sample	Plastic related	Use	Toxicity
94108-97-1	[2-[2,2-bis(prop-2-enoyloxymethyl)butoxymethyl]-2-(prop-2-enoyloxymethyl)butyl] prop-2-enoate	PP 1, PP 5b, PP 5a	yes (B)	Colorant, monomer (B)	Irritant (C). Not classified as of potential concern (B)
1541-67-9	lauryldiethanolamine	PP 3, PS 3	yes (C)	Flame retardant, PUR-resin forming composition, propylene resin composition (C)	Irritant, toxic to aquatic life, genotoxic (C)
1733-93-3	2-[2-dodecoxyethyl(2-hydroxyethyl)amino]ethanol	PP 3, PS 3	tentative	Cosmetics, antistatic agent, surfactant (C)	-
10213-78-2	2-[2-hydroxyethyl(octadecyl)amino]ethanol	PP 4	yes (B)	Antistatic agent, emulsifying, lubricants, processing aids (B, C)	Irritant, corrosive, toxic to aquatic life (C). Persistant and bioaccumulative (B)
52497-24-2	2-[2-hydroxyethyl(octadecyl)amino]ethyl octadecanoate	PP 4	yes (B)	Antistatic agent, FCA (B, C)	Corrosive, irritant (C). Not classified as of potential concern (B)
18924-66-8	2,2'-(Tetradecylimino)diethanol	PP 4	yes (C)	Antistatic agent, PUR resinforming, composition, PS resin particles (B, C)	Corrosive, irritant (C). Not classified as of potential concern in (B). AR antagonist, ERα, PPARγ and PXR agonist (D)
14174-09-5	2,5,8,11,18,21,24,27-octaoxatricyclo[26.4.0.012,17]dotriaconta-1(32),12,14,16,28,30-hexaene	PP 4	tentative	Adhesive, polymers for use as anion exchange membranes (C)	Irritant (C)
23681-60-9	tris(4-anilinophenyl)methanol	PP 5a, PP 5b	-	Purification of triphenylmethane compounds (C)	-

CAS	Name	High abundance in sample	Plastic related	Use	Toxicity
82799-44-8	2,4-diethylthioxanthen-9-one	PP 5a, PP 5b	yes (B)	Adhesive, colorant, filler, other processing aids (B)	Not classified as of potential concern (B)
68966-33-6	(4-aminophenyl)-bis(4-anilinophenyl)methanol	PP 5a, PP 5b	-	-	-
4687-94-9	[2-hydroxy-3-[4-[2-[4-(2-hydroxy-3-prop-2-enoyloxypropoxy)phenyl]propan-2-yl]phenoxy]propyl] prop-2-enoate	PP 5a, PP 5b	yes (B)	Monomer (B)	Irritant (C). Not classified as of potential concern (B)
29649-48-7	N-[5-[2-(2,5-dioxopyrrolidin-1-yl)ethyl-ethylamino]-2-[(4-nitrophenyl)diazenyl]phenyl]acetamide	PP 5a, PP 5b	-	-	-
-	2-[[4-[(4-aminophenyl)-(4-anilinophenyl)methylidene]cyclohexa-2,5-dien-1-ylidene]amino]benzenesulfonic acid	PP 5b	-	-	-
119313-12-1	2-benzyl-2-(dimethylamino)-1-(4-morpholin-4-ylphenyl)butan-1-one	PP 5a, PP 5b	yes (B)	Colorant, catalyst, filler, lubricant, other processing aids (B)	Persistent, bioaccumulate, CMR, reprotoxic, toxic to aquatic life (B). AR antagonist, ERα, PPARγ and PXR agonist (D)
67989-24-6	(3-hydroxy-2,2-dimethylpropyl) (Z)-octadec-9-enoate	PS 1	tentative	Lubricants and lubricant additives (C)	-
64050-16-4	2-[2,4-bis(2-methylbutan-2-yl)phenoxy]ethyl prop-2-enoate	PS 3	-	-	-

CAS	Name	High abundance in sample	Plastic related	Use	Toxicity
3386-18-3	2-[2-[2-[2-[2-[2-[2-[2-(2-hydroxyethoxy)ethoxy]ethoxy]ethoxy]ethoxy]ethoxy]ethoxy]ethoxy]ethanol	PS 4	yes (C)	Processing aids and additives, coating (C)	Irritant (C)
611-92-7	1,3-dimethyl-1,3-diphenylurea	PS 4	-	-	Irritant, toxic to aquatic life (C)
5617-32-3	2-[2-[2-[2-[2-[2-(2-hydroxyethoxy)ethoxy]ethoxy]ethoxy]ethoxy]ethoxy]ethanol	PS 4	tentative	Processing aids and additives (C)	
17598-96-8	2-[2-[2-[2-[2-[2-[2-[2-[2-[2-[2-(2-hydroxyethoxy)ethoxy]ethoxy]ethoxy]ethoxy]ethoxy]ethoxy]ethoxy]ethoxy]ethoxy]ethoxy]ethanol	PS 4	-	Cosmetics (C)	-
596-27-0	3,3-bis(4-hydroxy-3-methylphenyl)-2-benzofuran-1-one	PS 4	-	-	-
506-12-7	heptadecanoic acid	PS 5a	yes (B)	Surfactant, lubricant, other processing aids (B, C)	Not classified as of potential concern (B)
111-60-4	2-hydroxyethyl octadecanoate	PS 5a	yes (B)	Emulsifier, plasticizer, food additive (B, C)	Not classified as of potential concern (B)
71989-20-3	(2S)-5-amino-2-(9H-fluoren-9-ylmethoxycarbonylamino)-5-oxopentanoic acid	PS 5a	-	Peptide (C)	-
69701-99-1	(2-ethyl-2-methyl-1,3-dioxolan-4-yl)methyl prop-2-enoate	comp. 1	tentative	Ink, adhesives (C)	Irritant (C)
507-60-8	[8,14-dihydroxy-10,13-dimethyl-17-(6-oxopyran-3-yl)-3-[3,4,5-	comp. 1	-	rodenticide (C)	

CAS	Name	High abundance in sample	Plastic related	Use	Toxicity
	trihydroxy-6-(hydroxymethyl)oxan-2-yl]oxy-2,3,6,7,9,11,12,15,16,17-decahydro-1H cyclopenta[a]phenanthren-6-yl] acetate				
7441-32-9	3,6,13,16,23,26-hexaoxatetracyclo[26.2.2.28,11.218,21]hexatriaconta-1(31),8,10,18(34),19,21(33),28(32),29,35-nonaene-2,7,12,17,22,27-hexone	PET 1, PET 3, PET 4, PET 5, PET 6a	yes (B)	NIAS in PET (A)	-
58-18-4	(8R,9S,10R,13S,14S,17S)-17-hydroxy-10,13,17-trimethyl-2,6,7,8,9,11,12,14,15,16-decahydro-1H-cyclopenta[a]phenanthren-3-one	PET 2	-	Drug, steroid (C)	May cause cancer and reproductive toxicity (C). AR antagonist, ERα, PPARγ and PXR agonist (D)
-	(8S,9S,10S,13S,14S)-10,13-dimethyl-4,5,6,7,8,9,11,12,14,15,16,17-dodecahydro-3H-cyclopenta[a]phenanthren-1-ol	PET 2	-	Pharmaceutical (C)	-
62488-56-6	nona-2,4-dien-1-ol	PET 2	-	Flavoring agent in food, ink (C)	-
69686-05-1	1,8-dihydroxy-3-methyl-6-[(3R,4R,5R,6S)-3,4,5-trihydroxy-6-	PET 4	-	-	-

CAS	Name	High abundance in sample	Plastic related	Use	Toxicity
	methyloxan-2-yl]oxyanthracene-9,10-dione				
10287-53-3	ethyl 4-(dimethylamino)benzoate	PET 5	yes (B)	Colorant, filler, other processing aids (B)	Persistent and bioaccumulative, carcinogenic, mutagenic and reprotoxic, toxic to aquatic life (B, C) ERα, PPARγ and PXR agonist (D)
84-11-7	phenanthrene-9,10-dione	PET 5	tentative	Used in prod. of dyes, herbicides, pharmaceuticals, UV curable coatings and adhesives (C)	Irritant, cytotoxic (C)
18595-18-1	methyl 3-amino-4-methylbenzoate	PET 5	-	-	Irritant (C)
18814-21-6	1-[3,4-dihydroxy-5-(hydroxymethyl)oxolan-2-yl]-5-fluoropyrimidine-2,4-dione	PET 5	-	Pharmaceutical (C)	-
3147-75-9	Octrizole	PUR 1	yes (B)	UV absorber, antioxidant, UV light stabilizer in plastic, used in cosmetics, used in food contact substances (C)	Irritant, environmental hazard (C), not classified as of potential concern in (B), AR antagonist, ERα and PXR agonist (D)
115-86-6	Triphenyl phosphate	PUR 1, PUR 2	yes (B)	Plasticizer, other processing aids, FCA (A, C)	Environmental hazard, toxic to aquatic life, long-term hazard (C), Bioaccumulative, persistent, aquatic toxicity and EDC (B), AR antagonist, ERα, PPARγ and PXR agonist (D)
41556-26-7	bis(1,2,2,6,6-pentamethylpiperidin-4-yl) decanedioate	PUR 2	yes (B)	UV light absorber, stabilizer (C)	Toxic to aquatic life, irritant, persistant and bioaccumulative (B, C)

CAS	Name	High abundance in sample	Plastic related	Use	Toxicity
82919-37-7	1-O-methyl 10-O-(1,2,2,6,6-pentamethylpiperidin-4-yl) decanedioate	PUR 2	yes (B)	Antioxidant, stabilizer (C)	Toxic to aquatic life, irritant, persistant and bioaccumulative (B, C)
57834-33-0	ethyl 4-[(N-methylanilino)methylideneamino]benzoate	PUR 2	yes (B)	Colorant, filler, light stabilizer, other processing aids (B)	Toxic to aquatic life (long term hazard), harmful if swallowed, irritant (C), Not classified as of potential concern (B)
67845-92-5	8-(2,2,6,6-tetramethylpiperidine-4-carbonyl)oxyoctyl 2,2,6,6-tetramethylpiperidine-4-carboxylate	PUR 2	yes (B)	Polyester compositions (C)	-
5834-63-9	1,8,15,22-tetrazacyclooctacosane-2,9,16,23-tetrone	comp. 2	yes (A, B)		-
4266-66-4	1,8-diazacyclotetradecane-2,7-dione	comp. 2	yes (B)	Flame retardant, stabilizer (C)	Not classified as of potential concern (B)
96507-76-5	2-O-decyl 1-O-nonyl benzene-1,2-dicarboxylate	PVC 2	yes (C)	Plasticizer (C)	-
-	[(3R,4S,5S,6R)-3,4,5-trihydroxy-6-(hydroxymethyl)oxan-2-yl] (Z)-octadec-9-enoate	PVC 3	-	Alkyl glycoside-based micellar thickeners for surfactant systems (C)	-
56149-06-5	2,3-diacetyloxypropyl docosanoate	PVC 3	-	-	-
92739-54-3	10-dodecoxy-10-oxodecanoic acid	PVC 4	tentative	In inks containing crystalline polyesters (C)	-

S2.4 Limitations of the chemical analysis and identifications.

It should be noted that the number of chemicals reported here is not comprehensive as the measurements were only performed using positive ionization mode. This means that negatively ionizable compounds were not detected, possibly leading to an underestimation of the actual number of chemicals present. Yet, the largest fraction of plastic related chemicals is positively ionizable.[13] One-third of the samples analyzed had previous food content, which can alter the chemical composition to varying extents, as demonstrated by a comparison of three products analyzed both with and without previous food content. This could result in an overestimation; however, no general trend of higher numbers was observed in samples with previous food content. Furthermore, the four samples with the highest number of features did not have previous food content.

The low identification rate, as achieved in this study, is a known challenge, especially with liquid chromatographic methods. The scarcity of publicly available information on plastic chemicals paired with technical challenges of identification of LC-MS data hinders a more comprehensive identification.[14] While there is much uncertainty regarding the use of chemicals in plastics, about 44% of the top ten most abundant features in our samples were not clearly related to plastics based on the sources we consulted. These chemicals may have originated from the food content, be formed or introduced during plastic production (e.g., NIAS), are used in plastic production but not known to the public, or simply be misidentified.[5,15,16]

In another study which used a nontarget liquid chromatography coupled to high-resolution mass spectrometry,[17] 76% of the identified chemicals in food packaging were classified as NIAS. In accordance with this, Geueke et al. reported that 65% of chemicals in food contact materials, including plastics, are not listed for their use in food contact applications.[18] Nonetheless, 56% of the ten most abundant features were identified as chemicals probably used in plastics, indicating the suitability of this nontarget approach.

S2.5 Receptor activity of the tentatively identified chemicals retrieved from ToxCast

For prioritization of the chemicals, we used a ranking system to identify the most abundant chemicals in the samples as a proxy for their concentration and their potency of receptor activity

according to ToxCast (Table S9). To do so, we calculated the ratio of the chemical's AC_{50} over its abundance. Within the highest-ranking chemicals, we found the plastic related chemicals triphenyl phosphate (CAS 115-86-6), which is the highest ranking PXR (AC_{50} = 0.7 μM) and PPARγ (AC_{50} = 0.003 μM) agonist and was detected in ten samples made of six polymers or octrizole (CAS 3147-75-9, 2nd rank for PXR, AC_{50} = 1.0 μM) and tributyl 2-acetyloxypropane1,2,3-tricarboxylate (CAS 77-90-7, 3rd rank for PPARγ, AC_{50} = 7.4 μM). The ERα agonist with the lowest AC_{50}/abundance ratio is 4-dodecyl phenol (CAS 104-43-8, AC_{50} = 0.08 μM) which was detected in six FCAs (LDPE 8, PUR 1, PVC 3, 4, and comp. 1 and 2) and is an additive in lubricants and fuels. The highest-ranking anti-androgenic compound was cyclohexanone oxime (CAS 100-64-1, AC_{50} = 0.7 μM), a plastic-associated chemical detected in three FCAs (PUR 1, PVC 4 and comp. 2). Further, looking at high-potency plastic chemicals which were detected in more than six samples of different polymers, the plasticizer triethylene glycol (CAS 112-27-6, PXR AC_{50} = 0.11 μM) and tetraethylene glycol, (CAS 112-27-6, PPARγ AC_{50} = 0.06 μM) are notable. These compounds were detected in ten FCAs made of four polymers (LDPE 2, 4, 7, HDPE 3, comp. 1, PET 1, 5, PP 4, PS 4 and 5b) and in eight FCAs covering four polymers and the composites (LDPE 4, 7, 8, PP 5b, PS 5b, PVC 4, comp. 1 and 2), respectively. Both tetraethylene glycol and triethylene glycol were also identified as AR antagonists.

Table S9. PXR, PPARγ, ERα agonists and AR antagonists tentatively identified in the plastic extracts. Compounds ranked based on AC_{50}/peak area per receptor. First 20 compounds per receptor are listed for others see Table S15.

CAS	Rank	AC_{50} [µM]	Highest peak	Ratio AC_{50}/peak	Detected in samples
**PXR**					
115-86-6	1	0.70	542944	1.30E-06	PUR_1, PUR_2, PVC_2, LDPE_4, LDPE_7, comp_1, PET_4, PET_5, PP_5b, PS_4
3147-75-9	2	1.04	607970	1.71E-06	PUR_1, PUR_2, PET_2
77-90-7	3	0.85	184096	4.63E-06	comp_2, LDPE_2, PS_5b
791-28-6	4	0.70	132896	5.28E-06	PUR_1, PUR_2, comp_2, PP_5b, PP_5a
59227-89-3	5	1.41	191304	7.36E-06	PUR_1, comp_2, PVC_3, PVC_4, LDPE_8, LDPE_5, LDPE_7, LDPE_2, LDPE_3, comp_1
112-27-6	6	0.11	2626	4.37E-05	LDPE_4, LDPE_7, HDPE_3, LDPE_2, comp_1, PET_1, PET_5, PP_4, PS_4, PS_5b
104-43-8	7	1.45	31244	4.64E-05	PUR_1, comp_2, PVC_3, PVC_4, LDPE_8, comp_1
119313-12-1	8	1.63	31445	5.18E-05	LDPE_4, LDPE_7, PP_1, PP_5b, PP_5a
18924-66-8	9	2.22	39595	5.60E-05	LDPE_7, PP_4
4986-89-4	10	0.64	6559	9.81E-05	PP_1, PP_5b, PP_5a, PS_1
124-07-2	11	0.09	548	1.68E-04	PUR_1, PUR_2, PVC_1, PVC_3, PVC_4
3179-80-4	12	4.15	15416	2.69E-04	comp_2
68-26-8	13	0.30	952	3.14E-04	LDPE_4, LDPE_3, comp_1,
94-62-2	14	1.26	1989	6.34E-04	LDPE_4, LDPE_7, comp_1, PET_6b
75330-75-5	15	1.66	2163	7.67E-04	PVC_1
117-81-7	16	9.14	10032	9.11E-04	PVC_2
78-51-3	17	0.99	984	1.00E-03	LDPE_4
55179-31-2	18	1.16	915	1.27E-03	PUR_2, PVC_4,
42978-66-5	19	2.83	1926	1.47E-03	LDPE_8, comp_1, PP_5b, PP_5a,
6707-60-4	20	33.24	17349	1.92E-03	PUR_1, PUR_2, comp_2, PVC_3, PVC_4, LDPE_8, comp_1, PET_2, PS_5b
**PPARγ**					
115-86-6	1	0.003	542944	5.55E-09	PUR_1, PUR_2, PVC_2, LDPE_4, LDPE_7, comp_1, PET_4, PET_5, PP_5b, PS_4
87-62-7	2	0.014	5892	2.43E-06	PUR_2, PVC_3, PVC_4
77-90-7	3	7.39	184096	4.01E-05	comp_2, LDPE_2, PS_5b
59227-89-3	4	7.87	191304	4.12E-05	PUR_1, comp_2, PVC_3, PVC_4, LDPE_8, LDPE_5, LDPE_7, LDPE_2, LDPE_3, comp_1
112-60-7	5	0.06	745	8.40E-05	comp_2, PVC_4, LDPE_8, LDPE_4, LDPE_7, comp_1, PP_5b, PS_5b

CAS	Rank	AC_{50} [µM]	Highest peak	Ratio AC_{50}/peak	Detected in samples
4986-89-4	6	1.78	6559	2.71E-04	PP_1, PP_5b, PP_5a, PS_1
142-18-7	7	13.01	25094	5.19E-04	PUR_1, PUR_2, comp_2, PVC_3, PVC_4, LDPE_4, PET_2
105-95-3	8	92.22	99278	9.29E-04	PVC_3
119313-12-1	9	42.25	31445	1.34E-03	LDPE_4, LDPE_7, PP_1, PP_5b, PP_5a
94-09-7	10	40.96	17969	2.28E-03	PUR_1, PUR_2, PVC_3, PVC_4, LDPE_4
117-81-7	11	25.99	10032	2.59E-03	PVC_2
143-07-7	12	10.05	3872	2.60E-03	comp_2, PVC_3, PVC_4, LDPE_4, LDPE_5, LDPE_7, LDPE_2, LDPE_3, comp_1
58846-77-8	13	21.51	8029	2.68E-03	comp_2, PVC_1, PVC_3, PVC_4, LDPE_7, PS_4
104-43-8	14	89.04	31244	2.85E-03	PUR_1, comp_2, PVC_3, PVC_4, LDPE_8, comp_1
6707-60-4	15	63.93	17349	3.68E-03	PUR_1, PUR_2, comp_2, PVC_3, PVC_4, LDPE_8, comp_1, PET_2, PS_5b
1541-67-9	16	66.65	13092	5.09E-03	LDPE_7, PP_3, PS_3
3179-80-4	17	85.37	15416	5.54E-03	comp_2
8004-87-3	18	9.38	1128	8.32E-03	PP_5b, PP_5a
42978-66-5	19	17.29	1926	8.98E-03	LDPE_8, comp_1, PP_5b, PP_5a
16219-75-3	20	24.07	2636	9.13E-03	PVC_1, PVC_3, PVC_4
**ERα**					
104-43-8	1	0.08	31244	2.70E-06	PUR_1, comp_2, PVC_3, PVC_4, LDPE_8, comp_1
521-17-5	2	0.08	11309	7.12E-06	PUR_1, PVC_1, PVC_2, PVC_3, PVC_4
115-86-6	3	7.98	542944	1.47E-05	PUR_1, PUR_2, PVC_2, LDPE_4, LDPE_7, comp_1, PET_4, PET_5, PP_5b, PS_4
123-34-2	4	0.06	3839	1.67E-05	comp_2, PVC_3, PVC_4
119313-12-1	5	0.96	31445	3.06E-05	LDPE_4, LDPE_7, PP_1, PP_5b, PP_5a
3147-75-9	6	31.55	607970	5.19E-05	PUR_1, PUR_2, PET_2
25564-22-1	7	0.06	205	3.01E-04	LDPE_7
57-11-4	8	2.30	2043	1.12E-03	PVC_3, PVC_4, LDPE_8
75330-75-5	9	7.09	2163	3.28E-03	PVC_1
102-76-1	10	39.31	7900	4.98E-03	PUR_1, PVC_3, PVC_4
67-73-2	11	4.83	864	5.59E-03	PP_1, PP_5b, PP_5a
117-81-7	12	59.54	10032	5.94E-03	PVC_2
65-85-0	13	69.32	11145	6.22E-03	PUR_1, PVC_2, comp_1, PP_5a, PS_4, PS_5a, PS_5b
135-19-3	14	5.70	767	7.43E-03	PP_1, PS_3, PS_4, PS_5a, PS_5b
87-51-4	15	13.96	1843	7.58E-03	comp_2

CAS	Rank	AC_{50} [µM]	Highest peak	Ratio AC_{50}/peak	Detected in samples
4940-11-8	16	8.83	976	9.05E-03	PUR_1, PUR_2, comp_2, PVC_4
620-40-6	17	3.98	428	9.29E-03	PUR_1
1224-92-6	18	18.53	1687	1.10E-02	LDPE_4, LDPE_5, LDPE_6, LDPE_7, LDPE_2, LDPE_3, comp_1, PP_1, PP_5b, PP_5a, PS_4
21145-77-7	19	4.80	299	1.60E-02	PVC_3, PVC_4
57-11-4	20	2.30	143	1.61E-02	PE_LD_44, LDPE_4, LDPE_6, PE_LP_10, PE_LP_15, PS_4, PS41a
**AR**					
100-64-1	1	0.69	19747	3.50E-05	PUR_1, comp_2, PVC_4
115-86-6	2	25.28	542944	4.66E-05	PUR_1, PUR_2, PVC_2, LDPE_4, LDPE_7, comp_1, PET_4, PET_5, PP_5b, PS_4
3147-75-9	3	31.39	607970	5.16E-05	PUR_1, PUR_2, PET_2
59227-89-3	4	42.55	191304	2.22E-04	PUR_1, comp_2, PVC_3, PVC_4, LDPE_8, LDPE_5, LDPE_7, LDPE_2, LDPE_3, comp_1
87-62-7	5	1.62	5892	2.75E-04	PUR_2, PVC_3, PVC_4
18924-66-8	6	12.48	39595	3.15E-04	LDPE_7, PP_4
94-09-7	7	6.07	17969	3.38E-04	PUR_1, PUR_2, PVC_3, PVC_4, LDPE_4
4986-89-4	8	3.15	6559	4.80E-04	PP_1, PP_5b, PP_5a, PS_1
104-43-8	9	16.22	31244	5.19E-04	PUR_1, comp_2, PVC_3, PVC_4, LDPE_8, comp_1
99-96-7	10	0.60	1102	5.48E-04	PVC_1, PVC_2
75-07-0	11	1.15	1506	7.66E-04	PVC_3, PVC_4
96-41-3	12	0.27	305	8.72E-04	comp_2, PVC_3
78-51-3	13	0.89	984	9.00E-04	LDPE_4
495-18-1	14	40.58	40617	9.99E-04	PUR_1, PUR_2
119313-12-1	15	31.86	31445	1.01E-03	LDPE_4, LDPE_7, PP_1, PP_5b, PP_5a
3055-99-0	16	5.86	5544	1.06E-03	comp_2
3179-80-4	17	16.36	15416	1.06E-03	comp_2
8004-87-3	18	1.38	1128	1.23E-03	PP_5b, PP_5a
75330-75-5	19	3.57	2163	1.65E-03	PVC_1
1541-67-9	20	28.60	13092	2.18E-03	LDPE_7, PP_3, PS_3

Table S10. Number of known, active chemicals detected in the samples compared to the experimentally derived effect concentrations.

Sample	PXR agonists/ antagonists	EC_{20} [mg well^{-1}]	PPARg agonists/ antagonists	EC_{20} [mg well^{-1}]	ERa agonists/ antagonists	EC_{20} [mg well^{-1}]	AR antagonists/ agonists	EC_{50} [mg well^{-1}]
HDPE 1	0/0	0.26	0/0	0	0/0	0	0/0	0
HDPE 2	0/0	0	0/0	0	0/0	0	0/0	0
HDPE 3	3/0	0.15	1/0	1.03	2/0	0	1/0	0
HDPE 4	1/0	0	0/0	0	0/1	0	2/0	0
LDPE 1	1/0	0	1/0	0	0/0	0	0/0	0
LDPE 2	9/0	0.06	2/0	0.46	4/1	0	3/1	0.75
LDPE 3	8/0	0.12	4/0	0.47	3/1	0	3/1	0.52
LDPE 4	20/0	0.13	6/1	1.10	10/4	0	11/4	0
LDPE 5	5/0	0.06	2/0	0.21	1/1	0	1/0	0.41
LDPE 6	4/0	0.11	0/0	0.42	2/2	0	4/1	1.06
LDPE 7	25/0	0.07	7/1	0.59	15/4	0.15	9/4	0.73
LDPE 8	8/0	0	5/1	0.37	6/1	0	5/1	0
PET 1	4/0	0.21	1/0	0	0/1	0	5/1	0
PET 2	8/1	0.19	4/0	0	6/0	0	2/1	0
PET 3	0/0	0.73	0/0	0	0/0	0	0/0	0
PET 4	2/0	0	3/1	0	1/0	0	2/0	0
PET 5	4/0	0.78	2/1	0	3/0	0	4/0	0
PET 6a	0/0	0.54	0/0	0	0/0	0	0/0	0
PET 6b	2/0	0.15	0/0	0	1/0	0	1/0	0
PP 1	13/0	0.25	6/0	0	8/4	0	5/0	0
PP 2	0/0	1	0/0	0	1/0	0	0/0	0
PP 3	2/0	1	2/0	0	0/1	0	2/0	0
PP 4	4/0	0.37	4/0	0	3/2	0	4/1	0
PP 5a	10/0	0.16	6/1	0	6/4	1	7/4	0
PP 5b	6/0	0.22	5/0	0	4/3	0.00	5/4	0
PS 1	2/0	0.39	1/0	0	0/1	0.81	2/0	0
PS 2	0/0	0.60	0/0	0	0/0	0	0/0	0
PS 3	4/0	0.48	3/0	0	2/2	0.90	3/0	0
PS 4	13/1	0.15	5/1	0	9/4	0.65	10/3	0
PS 5a	5/0	0.16	3/0	0	3/1	0.21	3/0	1.13
PS 5b	7/1	0.07	4/0	0	5/1	0.09	4/0	0.67
PUR 1	28/2	0	6/2	0	18/3	0	15/9	0
PUR 2	30/1	0.01	7/3	0	14/5	0	18/7	0
PVC 1	10/0	0.11	2/0	0.17	6/0	0	4/3	0.26
PVC 2	6/0	0.51	5/0	1.14	6/0	0	5/2	0
PVC 3	26/4	0.15	11/4	0.58	15/6	0	20/7	0
PVC 4	26/6	0.01	10/3	0.15	16/10	0	22/7	0.29
comp. 1	22/1	0.11	10/1	0.66	14/2	0	8/5	0
comp. 2	31/6	0.01	10/2	0.53	12/8	0	16/5	0.88

S2.6 Predictors of receptor activity

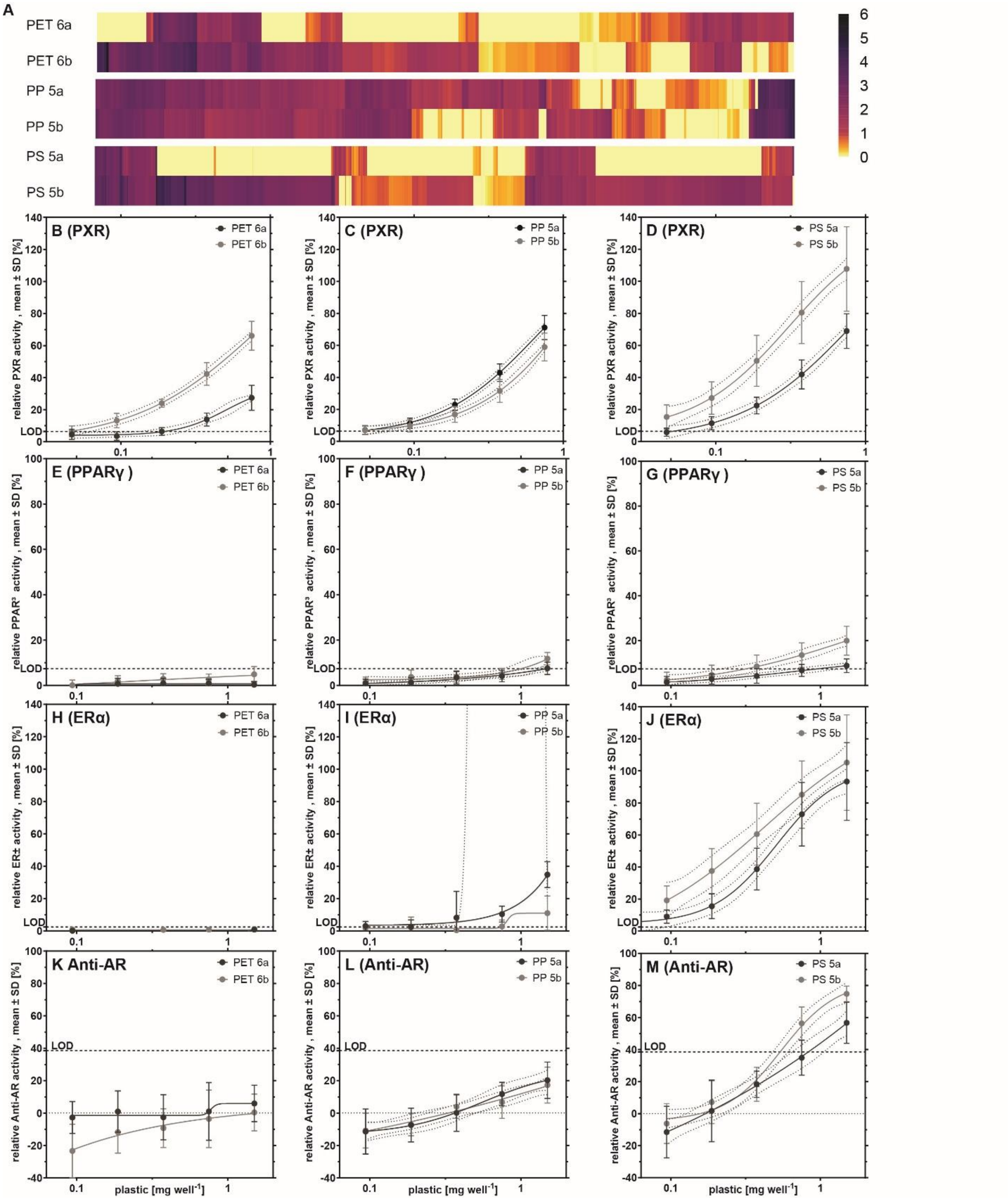

Figure S7. Comparison of samples without (a) and with (b) previous food content on chemical composition (A) and (B–M) receptor activity. Bioassay data are derived from at least three independent experiments, with four technical replicates per concentration (n ≥ 12).

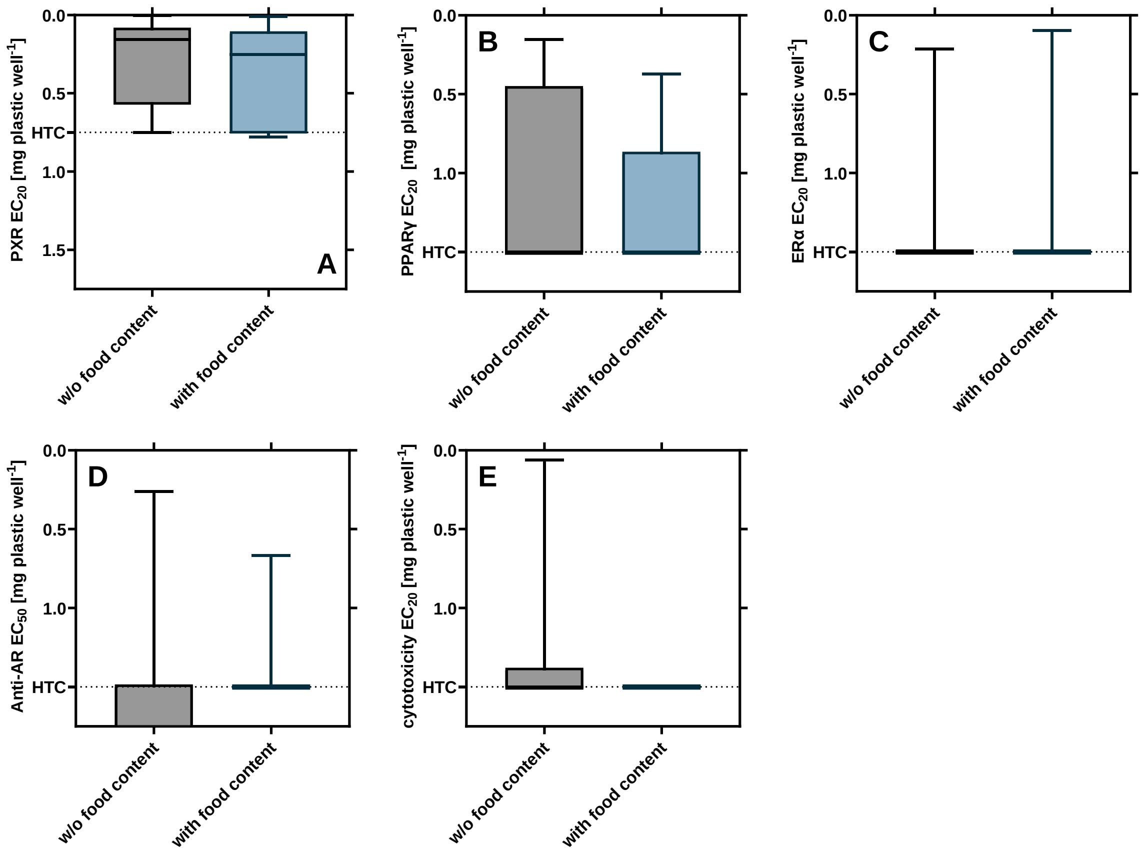

Figure S8**.** Effect of previous food content on receptor activity and cytotoxicity. $EC_{20/50}$ values calculated from a minimum of three independent experiments with four technical replicates per concentration ($n \geq 12$). Mann-Whitney tests do not indicate significant differences ($p > 0.05$).

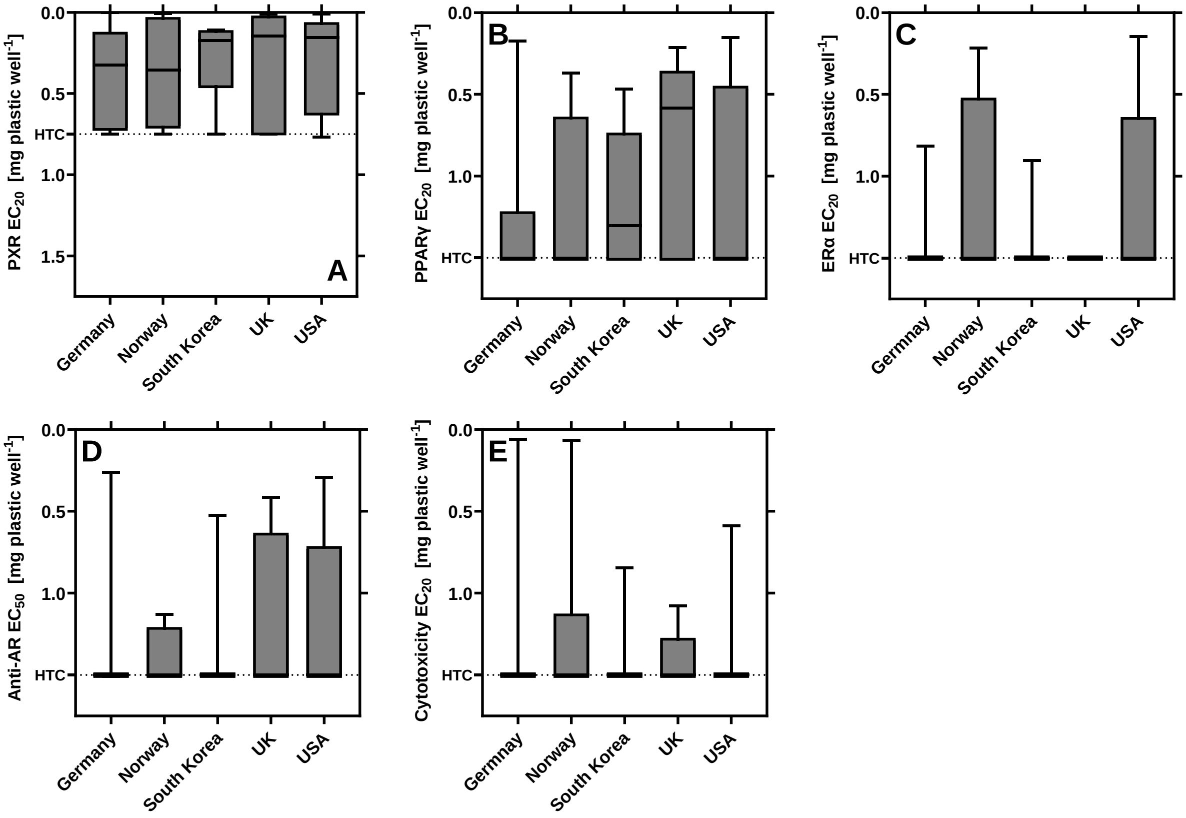

Figure S9. Impact of country of purchase on receptor activity and cytotoxicity. $EC_{20/50}$ values calculated from a minimum of three independent experiments with four technical replicates per concentration ($n \geq 12$). Kruskal Wallis tests and Dunn's multiple comparison tests do not indicate significant differences ($p > 0.05$).

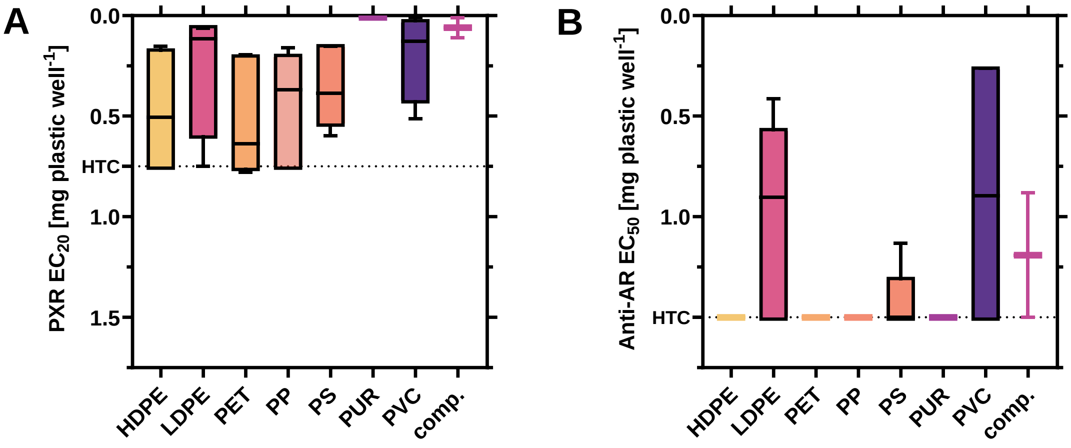

Figure S10. Impact of the polymer type on receptor activity at (A) PXR, (B) Anti-AR. $EC_{20/50}$ calculated from at least three independent experiments with four technical replicates per concentration ($n \geq 12$). Kruskal-Wallis tests with Dunn's multiple comparison tests do not indicate significant differences ($p > 0.05$).

S2.7 Partial least squares regression

Table S11**.** Performance of the PLS regression of chemical features (occurrence and abundance) and receptor activity (normalized $EC_{20/50}$ values) after iterative filtering steps excluding features with VIP <0.8. PLS regression with food contact articles made of PE, PET, PP and PS. Calculated (Cal), cross-validated (CV), cumulative explained variance (cumexpvar) of the chemical features (X) and receptor activity (Y), determination coefficient (R^2), root mean squared errors (RMSE), slope for predicted vs. measured values (slope), bias for prediction vs. measured values (bias), ratio of standard deviation of response values to standard error of prediction (RPD).

Model	N selected components	N features		X cumexpvar	Y cumexpvar	R^2	RMSE	Slope	Bias	RPD
**PXR**										
0	3	2993	Cal	46.0	76.9	0.77	17.3	0.769	0	2.11
			Cv	NA	NA	0.38	28.3	0.565	-1.44	1.29
1	4	2147	Cal	69.89	88.93	0.89	12.0	0.889	0	3.05
			Cv	NA	NA	0.62	22.2	0.766	-0.34	1.65
2	4	1761	Cal	67.80	89.99	0.90	11.4	0.9	0	3.21
			Cv	NA	NA	0.62	22.3	0.772	0.40	1.64
3	4	1583	Cal	65.56	90.39	0.90	11.2	0.904	0	3.28
			Cv	NA	NA	0.64	21.7	0.791	0.21	1.68
4	4	1544	Cal	63.03	90.54	0.91	11.1	0.905	0	3.3
			Cv	NA	NA	0.64	21.5	0.798	0.25	1.7
5	4	1536	Cal	62.39	90.57	0.91	11.1	0.906	0	3.31
			Cv	NA	NA	0.64	21.5	0.798	0.28	1.7
**6**	**4**	**1533**	**Cal**	**62.25**	**90.58**	**0.91**	**11.1**	**0.906**	**0**	**3.31**
			**Cv**	**NA**	**NA**	**0.64**	**21.5**	**0.798**	**0.28**	**1.7**
**PPARγ**										
0	1	2993	Cal	25.53	69.80	0.70	17.4	0.698	0	1.85
			Cv	NA	NA	0.58	20.6	0.612	-0.91	1.56
1	1	1232	Cal	81.48	69.13	0.69	17.6	0.691	0	1.83
			Cv	NA	NA	0.62	19.5	0.696	-0.73	1.65
2	2	836	Cal	86.88	85.11	0.85	12.2	0.851	0	2.63
			Cv	NA	NA	0.68	17.8	0.765	0.70	1.81
3	2	756	Cal	85.00	85.45	0.86	12.1	0.855	0	2.66
			Cv	NA	NA	0.71	17.0	0.772	0.73	1.89
4	2	734	Cal	84.51	85.47	0.86	12.1	0.855	0	2.67
			Cv	NA	NA	0.72	16.8	0.772	0.75	1.91
5	2	731	Cal	84.20	85.48	0.86	12.0	0.855	0	2.67
			Cv	NA	NA	0.72	16.8	0.773	0.75	1.92
**6**	**2**	**729**	**Cal**	**84.23**	**85.48**	**0.86**	**12.0**	**0.855**	**0**	**2.67**
			**Cv**	**NA**	**NA**	**0.72**	**16.8**	**0.773**	**0.75**	**1.92**

Model	N selected components	N features		X cumexpvar	Y cumexpvar	R^2	RMSE	Slope	Bias	RPD
**ERα**										
0	4	2993	Cal	53.21	94.34	0.94	7.2	0.943	0	4.27
			Cv	NA	NA	0.37	24.0	0.473	-2.11	1.28
1	5	1005	Cal	88.82	98.80	0.99	3.3	0.988	0	9.29
			Cv	NA	NA	0.71	16.3	0.665	1.85	1.89
2	3	596	Cal	74.99	96.87	0.97	5.3	0.969	0	5.74
			Cv	NA	NA	0.83	12.4	0.819	1.02	2.47
3	3	419	Cal	77.93	96.70	0.97	5.5	0.967	0	5.59
			Cv	NA	NA	0.87	10.7	0.872	0.23	2.86
4	3	368	Cal	75.18	96.63	0.97	5.5	0.966	0	5.54
			Cv	NA	NA	0.89	10.2	0.923	-0.09	2.99
5	3	349	Cal	74.58	96.52	0.97	5.6	0.965	0	5.44
			Cv	NA	NA	0.89	10.2	0.922	0.06	3
6	3	336	Cal	74.38	96.74	0.97	5.4	0.967	0	5.62
			Cv	NA	NA	0.90	9.7	0.915	0.19	3.16
7	3	333	Cal	73.56	96.83	0.97	5.4	0.968	0	5.7
			Cv	NA	NA	0.90	9.6	0.913	0.24	3.18
**8**	**3**	**332**	**Cal**	**73.61**	**96.81**	**0.97**	**5.4**	**0.968**	**0**	**5.69**
			**Cv**	**NA**	**NA**	**0.90**	**9.7**	**0.914**	**0.23**	**3.15**
**Anti-AR**										
0	1	2993	Cal	24.39	72.22	0.72	13.2	0.722	0	1.93
			Cv	NA	NA	0.55	16.8	0.584	-0.62	1.51
1	1	1043	Cal	87.16	68.63	0.69	14.0	0.686	0	1.81
			Cv	NA	NA	0.62	15.4	0.659	-0.28	1.65
2	4	751	Cal	90.90	97.62	0.98	3.9	0.976	0	6.58
			Cv	NA	NA	0.66	14.6	0.77	0.27	1.74
3	5	678	Cal	92.72	98.91	0.99	2.6	0.989	0	9.73
			Cv	NA	NA	0.73	13.0	0.795	0.74	1.96
4	5	662	Cal	92.38	98.97	0.99	2.5	0.99	0	9.99
			Cv	NA	NA	0.74	12.9	0.8	0.76	1.98
**5**	**5**	**661**	**Cal**	**92.22**	**98.98**	**0.99**	**2.5**	**0.99**	**0**	**10.05**
			**Cv**	**NA**	**NA**	**0.74**	**12.9**	**0.8**	**0.77**	**1.98**

Table S12. Performance of the PLS regression of chemical features (abundance) and receptor activity (normalized $EC_{20/50}$ values) detected in PE, PET, PP and PS FCAs and comparison of cross-validated (CV) root mean squared errors (RMSE) and CV determination coefficient (R^2) of the initial model 0 and optimized model (VIP filtered). Number of features included in the optimized model and corresponding values for the validation based on 500 iterations with randomly chosen variables. Model 0 included 2993 features.

Activity	RMSE CV model 0	RMSE CV optimized model	R^2 CV optimized model	Nr features optimized model	PLSR No	RMSE CV random		R^2 CV random	
						min	max	min	max
PXR	28	22	0.64	1533 (51%)	7	30	31	0.26	0.32
PPARγ	21	17	0.72	729 (24%)	6	20	22	0.53	0.61
ERα	24	10	0.9	332 (11%)	9	29	37	-0.54	0.1
Anti-AR	17	13	0.74	661 (22%)	6	16	18	0.5	0.6

Table S13. AC_{50} values of tentatively identified compounds in the optimized PLS regression models positively correlated with the receptor activity.

PLS model	Compound Name	CAS	AC_{50}	Plastic related	Use
PXR	triethylene glycol	112-27-6	**0.1**	yes	plasticizer, solvent, fragrance
	piperine	94-62-2	**1.3**		food additive
PPARγ	tetradecanoic acid	544-63-8	118.2	yes	surfactant
	laurocapram	59227-89-3	7.9		cosmetics
ERα	triphenyl phosphate	115-86-6	7.31	yes	plasticizer, flame retardant
AR	1-dodecyl-2-pyrrolidinone	2687-96-9	81.5	tentative	surfactant

Supporting References

(1) Primpke, S.; Wirth, M.; Lorenz, C.; Gerdts, G. Reference Database Design for the Automated Analysis of Microplastic Samples Based on Fourier Transform Infrared (FTIR) Spectroscopy. *Anal. Bioanal. Chem.* **2018**, *410* (21), 5131–5141. https://doi.org/10.1007/s00216-018-1156-x.

(2) Kolde, R. Package "Pheatmap": Pretty Heatmaps. Version 1.0.12. *CRAN* **2019**.

(3) Larsson, J.; Godfrey, A. J. R.; Gustafsson, P.; Eberly, D. H.; Huber, E.; Privé, F. Package

‘Eulerr’: Area-Proportional Euler and Venn Diagrams with Ellipses. Version 7.0.0. *CRAN* **2022**.

(4) Zimmermann, L.; Bartosova, Z.; Braun, K.; Oehlmann, J.; Völker, C.; Wagner, M. Plastic Products Leach Chemicals That Induce In Vitro Toxicity under Realistic Use Conditions. *Environ. Sci. Technol.* **2021**, *55* (17), 11814–11823. https://doi.org/10.1021/acs.est.1c01103.

(5) Wiesinger, H.; Wang, Z.; Hellweg, S. An Extensive Overview of Plastic Monomers, Additives and Processing Aids. *Environ. Sci. Technol.* **2021**, *55* (13), 9339–9351. https://doi.org/10.1021/acs.est.1c00976.

(6) United States Environmental Protection Agency (US EPA). *CompTox Chemicals Dashboard*. https://comptox.epa.gov/dashboard/batch-search (accessed 2021-10-01).

(7) National Center for Biotechnology Information (NCBI). *PubChem Identifier Exchange Service*. https://pubchem.ncbi.nlm.nih.gov/idexchange/idexchange.cgi (accessed 2021-10-01).

(8) National Center for Biotechnology Information (NCBI). *Pub Chem. Open Chemistry Database.* https://pubchem.ncbi.nlm.nih.gov. (accessed 2022-03-01).

(9) Groh, K. J.; Backhaus, T.; Carney-Almroth, B.; Geueke, B.; Inostroza, P. A.; Lennquist, A.; Leslie, H. A.; Maffini, M.; Slunge, D.; Trasande, L.; Warhurst, A. M.; Muncke, J. Overview of Known Plastic Packaging-Associated Chemicals and Their Hazards. *Sci. Total Environ.* **2019**, *651*, 3253–3268. https://doi.org/10.1016/j.scitotenv.2018.10.015.

(10) Escher, B. I.; Stapleton, H. M.; Schymanski, E. L. Tracking Complex Mixtures of Chemicals in Our Changing Environment. **2020**, *392* (January), 388–392.

(11) Alygizakis, Nikiforos; Slobodnik, J. *S32 | REACH2017 | >68,600 REACH Chemicals*. https://doi.org/10.5281/zenodo.2653021.

(12) United States Environmental Protection Agency (US EPA). *ToxCast database (invitroDBv3).* https://www.epa.gov/chemical-research/generating-toxcast-data-chemical-lists#Downloadable Data (accessed 2022-03-01).

(13) Nerin, C.; Alfaro, P.; Aznar, M.; Domeño, C. The Challenge of Identifying Non-

Intentionally Added Substances from Food Packaging Materials: A Review. *Anal. Chim. Acta* **2013**, *775*, 14–24. https://doi.org/10.1016/j.aca.2013.02.028.

(14) Nerín, C.; Bourdoux, S.; Faust, B.; Gude, T.; Lesueur, C.; Simat, T.; Stoermer, A.; Van Hoek, E.; Oldring, P. Guidance in Selecting Analytical Techniques for Identification and Quantification of Non-Intentionally Added Substances (NIAS) in Food Contact Materials (FCMS). *Food Addit. Contam. Part A* **2022**, *39* (3), 620–643. https://doi.org/10.1080/19440049.2021.2012599.

(15) Muncke, J. Exposure to Endocrine Disrupting Compounds via the Food Chain: Is Packaging a Relevant Source? *Sci. Total Environ.* **2009**, *407* (16), 4549–4559. https://doi.org/10.1016/j.scitotenv.2009.05.006.

(16) Stricker, T.; Bonner, R.; Lisacek, F.; Hopfgartner, G. Adduct Annotation in Liquid Chromatography/High-Resolution Mass Spectrometry to Enhance Compound Identification. *Anal. Bioanal. Chem.* **2021**, *413* (2), 503–517. https://doi.org/10.1007/s00216-020-03019-3.

(17) Vera, P.; Canellas, E.; Nerín, C.; Dreolin, N.; Goshawk, J. The Migration of NIAS from Ethylene-Vinyl Acetate Corks and Their Identification Using Gas Chromatography Mass Spectrometry and Liquid Chromatography Ion Mobility Quadrupole Time-of-Flight Mass Spectrometry. *Food Chem.* **2022**, *366*, 130592. https://doi.org/10.1016/j.foodchem.2021.130592.

(18) Geueke, B.; Groh, K. J.; Maffini, M. V.; Martin, O. V.; Boucher, J. M.; Chiang, Y. T.; Gwosdz, F.; Jieh, P.; Kassotis, C. D.; Łańska, P.; Myers, J. P.; Odermatt, A.; Parkinson, L. V.; Schreier, V. N.; Srebny, V.; Zimmermann, L.; Scheringer, M.; Muncke, J. Systematic Evidence on Migrating and Extractable Food Contact Chemicals: Most Chemicals Detected in Food Contact Materials Are Not Listed for Use. *Crit. Rev. Food Sci. Nutr.* **2022**, *63* (28), 9425–9435. https://doi.org/10.1080/10408398.2022.2067828.
